# Nutritional Epigenetics of Vitamin Intake (A, B_1_, B_2_, B_3_, B_6_, B_12_, and E): Associations with Genome-Wide DNA Methylation

**DOI:** 10.3390/nu18152474

**Published:** 2026-07-30

**Authors:** Sadiye Aras, Jiajun Shi, Shuai Xu, Jie Wu, Wanqing Wen, Regina Courtney, Hui Cai, Xiao-Ou Shu, Wei Zheng, Jirong Long, Qiuyin Cai, Xiaofei Wang

**Affiliations:** 1 Department of Biological Sciences, Tennessee State University, Nashville, TN 37209, USA; saras@tnstate.edu; 2 Division of Epidemiology, Department of Medicine, Vanderbilt Epidemiology Center, Vanderbilt-Ingram Cancer Center, Vanderbilt University School of Medicine, Nashville, TN 37232, USA; jiajun.shi@vumc.org (J.S.); shuai.xu.1@vanderbilt.edu (S.X.); jie.wu@vumc.org (J.W.); wanqing.wen@vumc.org (W.W.); regina.courtney@vumc.org (R.C.); hui.cai@vumc.org (H.C.); xiao-ou.shu@vanderbilt.edu (X.-O.S.); wei.zheng@vanderbilt.edu (W.Z.); jirong.long@vanderbilt.edu (J.L.); qiuyin.cai@vanderbilt.edu (Q.C.)

**Keywords:** DNA methylation, nutritional epigenetics, vitamins, epigenome-wide association study, EWAS, SCCS, micronutrients

## Abstract

Background/Objectives: DNA methylation is a key epigenetic mechanism involved in gene regulation and chromatin stability and may be influenced by nutritional exposures. However, population-level evidence regarding associations between dietary vitamin intake and genome-wide DNA methylation remains limited. Methods: We investigated associations between dietary intake of vitamins A, B_1_, B_2_, B_3_, B_6,_ B_12_, and E and blood DNA methylation in 2030 participants from the Southern Community Cohort Study (SCCS). Dietary intake was assessed using a validated Food Frequency Questionnaire, and genome-wide DNA methylation was profiled using the Illumina Infinium MethylationEPIC v2.0 BeadChip. Epigenome-wide association analyses were conducted using robust linear regression models adjusted for demographic, lifestyle, and estimated cellular composition variables. Results: We identified 34 CpG sites associated with dietary intake of vitamins B_1_, B_2_, B_3_, B_6_, and E, at a false discovery rate-adjusted *p*-value (FDR_*p*) threshold of <0.1. The strongest association was observed between vitamin B_6_ intake and cg19831775 (Beta = −0.111, FDR_*p* = 0.015). No statistically significant associations were identified for vitamins A and B_12_. Most observed effects were modest, and associations were locus-specific rather than global. Race- and sex-stratified analyses demonstrated generally consistent directions of association, although statistical significance was attenuated in subgroup analyses. Conclusions: These findings provide suggestive evidence that dietary intake of vitamins B_1,_ B_2_, B_3_, B_6_ and E may be associated with subtle variation in DNA methylation at specific genomic loci.

## 1. Introduction

DNA methylation, marked by the covalent addition of a methyl group (-CH3) to cytosine residues at CpG dinucleotides, is a key epigenetic mechanism that controls gene expression and chromatin stability without changing the primary DNA sequence [1,2]. Abnormal methylation patterns, such as site-specific promoter hypermethylation and global hypomethylation, are linked to the development of cancer, cardiovascular disease, and neurodegenerative disorders [3,4,5]. During cellular differentiation, the methylome remains flexible and can respond to external influences throughout a person’s life. These adaptable epigenetic marks help maintain proper cell identity and react to environmental factors like diet, air pollution, psychosocial stress, and smoking [6,7,8]. Several vitamins may be associated with DNA methylation because they supply cofactors for one-carbon metabolism. S-adenosylmethionine (SAM), the universal methyl donor, is produced through a metabolic pathway that depends on B vitamins, including B_1_ (thiamine), B_2_ (riboflavin), B_3_ (niacin and nicotinamide), and B_6_ (pyridoxine) [9,10,11]. B vitamins are water-soluble and must be consumed daily. These vitamins are crucial for energy metabolism, supporting many physiological processes and cellular functions [12]. B_9_ folate plays a key role in one-carbon metabolism and methyl group donation, making it an important micronutrient in epigenetic regulation. Previous studies have linked folate intake and folic acid supplementation to altered DNA methylation and disease risk [13]. Ly et al. showed that high maternal folic acid intake can modify DNA methylation and increase tumor risk in offspring [14]. Overall, these findings support the role of folate in epigenetic modulation and disease susceptibility.

Beyond the B-complex lipid-soluble micronutrient, vitamin E (tocopherols and tocotrienols) is an antioxidant. It has been found to modulate cell signaling pathways, combating oxidative stress, providing anti-inflammation, anti-cancer, and anti-apoptosis effects [15]. Many of these effects are not apparent through defined pathways. It may protect the integrity of epigenetic marks. By embedding within the lipid bilayer of cell membranes, vitamin E neutralizes reactive oxygen species (ROS), thereby mitigating oxidative stress-induced damage to the methylome and supporting long-term neuroprotection and cardiovascular health [16]. Vitamin E was shown to be able to modulate promoter methylation of certain genes [17,18]. Whether vitamin E has a genome-wide effect on DNA methylation is unclear.

The public health implications of epigenetic modifications are profound, particularly regarding health disparities [19,20]. Evidence from studies in men with end-stage heart failure suggested that cardiac muscle DNA methylation is associated with racial and socioeconomic status [21]. Other aspects of health inequities observed across racial and socioeconomic lines may be driven by differential epigenetic responses to long-term dietary patterns and environmental stressors [7].

Prior research on the effect of some vitamins that are less apparently connected with DNA methylation, to our knowledge, has been limited by small sample sizes [8], few genomic loci [17,18,22], global methylation measurements without specific CpG site identification [23,24], and LINE-1 repetitive DNA [13]. Keshawarz and colleagues conducted a meta-analysis on the associations of vitamins E and C on DNA methylation based on several study populations [25]. This meta-analysis revealed that vitamin E is associated with a few hundred CpG site methylation changes. It is not clear whether these vitamin E associations hold in other populations, such as those recruited from the southeastern United States [26,27].

To address these critical gaps, we utilized data from the Southern Community Cohort Study (SCCS), a large-scale prospective cohort of predominantly low-income African American and European American adults in the southeastern United States. This approach offers new insights into diet-based strategies for reducing health disparities, employing the high-resolution Illumina Infinium MethylationEPIC v2.0 BeadChip, interrogating approximately 930,000 CpG sites with granular precision. By leveraging validated Food Frequency Questionnaires (FFQs) to assess the long-term, habitual intake of vitamins A, B_1_, B_2_, B_3_, B_6_, B_12,_ and E, we move beyond transient snapshots of nutrition to capture cumulative exposure. By applying a robust statistical framework within this historically underrepresented population, this research seeks to elucidate how specific micronutrients modulate epigenetic architecture, providing additional insight into potential relationships between nutrition and epigenetic variation in underserved populations.

**Figure 1:**
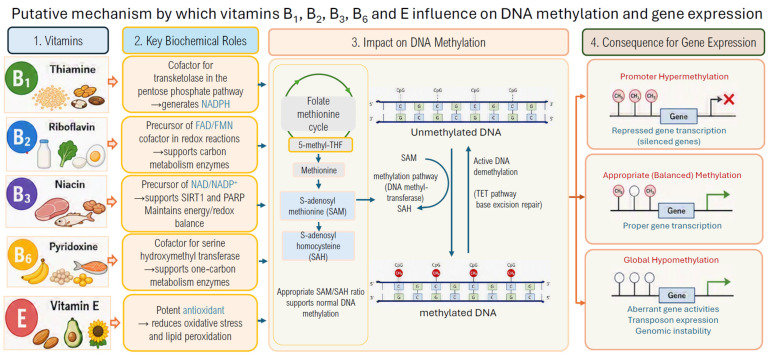
Diagram illustrating putative mechanisms by which vitamins B_1_, B_2_, B_3_, B_6_ and E modulate DNA methylation through one-carbon metabolism and TET demethylation pathways.

As illustrated in Figure 1, DNA methylation is an epigenetic modification in which a methyl group is added to the fifth carbon of cytosine (5-methylcytosine, 5-mC), primarily at CpG dinucleotides, often leading to transcriptional repression when present in gene promoter regions. Reversely, active DNA demethylation is initiated by Ten-eleven translocation (TET) enzymes (TET1, TET2, and TET3), which sequentially oxidize 5-mC to 5-hydroxymethylcytosine (5-hmC), 5-formylcytosine (5-fC), and 5-carboxylcytosine (5-caC), followed by base excision repair to restore unmodified cytosine [28]. In this proposed mechanism, vitamins B_1_, B_2_, B_3_, and B_6_ support one-carbon metabolism and provide essential cofactors that facilitate TET enzyme activity, whereas vitamin E reduces oxidative stress, preserving the cellular environment required for efficient epigenetic regulation. Together, these micronutrients modulate DNA methylation, alter chromatin accessibility, and shift gene transcription.

## 2. Materials and Methods

### 2.1. Study Population and Baseline Data Collection

The Southern Community Cohort Study (SCCS) is a prospective, population-based cohort of approximately 86,000 adults (aged 40–79 years) recruited between 2002 and 2009 across 12 southeastern U.S. states. Recruitment primarily occurred through Community Health Centers (CHCs) to ensure the inclusion of underserved populations, specifically African Americans and individuals of low socioeconomic status. At baseline, participants completed comprehensive questionnaires regarding demographics, medical history, and lifestyle factors. The cohort is characterized by a high burden of chronic conditions, including obesity (44%), hypertension (55%), and a history of smoking (45%) [29]. The SCCS was reviewed and approved by the institutional review boards at Vanderbilt University and Meharry Medical College. Written informed consent was obtained from all study participants.

### 2.2. Dietary Assessment

Long-term dietary intake was evaluated using a validated 104-item Food Frequency Questionnaire (FFQ) specifically calibrated for southeastern U.S. regional and racial dietary patterns [26]. Daily intakes of vitamins A, B_1_, B_2_, B_3_, B_6_, B_12_, and E were estimated using food composition databases from the USDA and the University of Minnesota Nutrition Coordinating Center. Nutrient intakes were energy-adjusted using the nutrient density method (amount per 1000 kcal). To account for sociocultural variations in diet, analysis utilized sex- and race-specific quartiles of intake [29,30].

### 2.3. Blood Collection and DNA Methylation

Baseline blood samples were provided by SCCS participants during enrollment at CHCs. Details of the blood collection and storage methods and laboratory procedures have been previously described [29,31]. Briefly, after blood draw, samples were immediately refrigerated and shipped cold overnight to the Vanderbilt University Medical Center Molecular Epidemiology Core Laboratory. Buffy coat samples were isolated and stored at −80 °C. DNA was extracted from buffy coats using Qiagen’s QIAamp DNA kits (Germantown, MD, USA) following manufacture’s instruction. Genome-wide DNA methylation was profiled using the Illumina Infinium MethylationEPIC v2.0 BeadChip (San Diego, CA, USA) at Vanderbilt’s VANTAGE core following Illumina’s protocols.

Raw DNA methylation array data were annotated with the Bioconductor IllimuniaHumanMetylationEPICv2anno.20a1.hg38 package [32]. IDAT files of CpG sites were converted into methylated and unmethylated signals assigned to methylation *β* values in a range between 0 (indicating unmethylated) and 1 (indicating fully methylated). Probes with detection *p* > 0.01 among more than three samples, low bead counts (*n* < 3), at non-CpG sites, or on sex chromosomes were excluded using the minfi [33] and ChAMP R packages (Version 2.42) [34]. DNA methylation levels were normalized using the single-sample normal exponential out-of-band background correction (ssnoob) [33] for background correction and dye-bias adjustment and using beta mixture quantile (BMIQ) normalization algorithm [35] to correct the differences in the distributions of beta values between Type I and Type II probes. Samples with >5% failed probes, without dietary vitamin intake information, or those diagnosed with lung cancer within two years after baseline survey were removed. Methylation *β* values across the replication probes were averaged. A total of 800,379 CpGs and 2030 samples were included in the final analysis. Circulating vitamin measurements were not available for the study population. Therefore, dietary intake estimates derived from a validated FFQ were used. While biomarkers may provide more objective assessments of short-term nutritional status, FFQs are intended to capture long-term habitual dietary intake, which was the exposure of interest in this study.

### 2.4. Statistical Analysis and Bioinformatics

Immune cell type proportions were imputed using FlowSorted.Blood.EPIC R package. Methylation M-values [36,37] were used as the primary outcome in this epigenome-wide association study (EWAS). Linear regression models with the sandwich correction were fitted to assess the association between vitamin intake and DNA methylation level at each CpG. All models were adjusted for age, sex, race, BMI, education level, annual household income, smoking status, alcohol intake (drinks per day), total energy intake, and estimated cellular composition (proportions of CD8T, CD4T, neutrophil, natural killer, and monocyte cells). Multiple tests were corrected using the Benjamini–Hochberg False Discovery Rate (FDR_*p*) method. Given the exploratory nature of EWAS analyses and the modest expected effect sizes for dietary exposures, an FDR_*p* threshold of <0.1 was used to identify suggestive associations. No independent replication cohort was available for validation of the observed associations.

## 3. Result

### 3.1. Characteristics of Participants from the Southern Community Cohort Study

Characteristics of participants from the Southern Community Cohort Study are presented in Table 1. The study included 2030 participants, of whom 54% were Black and 46% were male. The median age at baseline was 54 years (IQR: 48–61), with White participants being older than Black participants (*p* < 0.001). The cohort was characterized by generally low socioeconomic status, with 38.3% having less than a high school education and 64.1% reporting an annual household income below $15,000. Nearly 70% of participants were current smokers, and the median pack-years among ever smokers was 24.0 (IQR: 11.9–41.0). Median total energy intake was 2155 kcal/day, and the median Healthy Eating Index score was 55.2. Significant racial differences were observed for most demographic, lifestyle, and dietary variables, including vitamin intakes. Median intakes of vitamins B_1_, B_2_, B_3_, B_6_, and E differed significantly between Black and White participants (all *p* < 0.001), reflecting variation in dietary patterns within the cohort.

### 3.2. Vitamin Intake and DNA Methylation Associations

**Figure 2:**
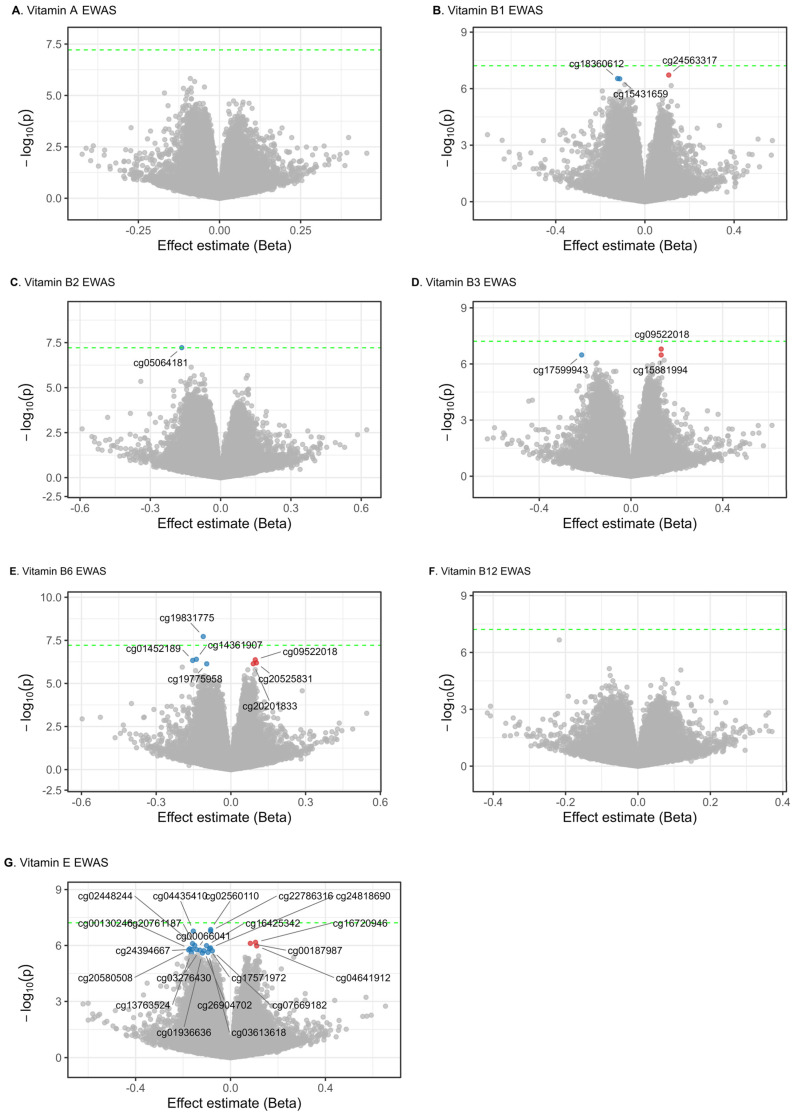
Volcano plots for epigenome-wide associations with respect to dietary intake of vitamins. Blue dots indicate negative associations, and red dots indicate positive associations at FDR_*p* < 0.10, while grey dots indicate not significant associations. The green dashed lines indicate epigenome-wide significance threshold at *p* value = 1.86 × 10^−8^.

Epigenome-wide association analyses identified multiple CpG sites associated with dietary vitamin intake (Table 2, Figure 2). Three CpG sites were associated with vitamin B_1_, one with vitamin B_2_, three with vitamin B_3_, seven with vitamin B_6_, and twenty-one with vitamin E. In total, there were 34 associated unique CpG sites. Among these 34 sites, cg09522018 appeared for both B_3_ and B_6_. The strongest association was observed for vitamin B_6_ at cg19831775 (effect size Beta = −0.111, FDR_*p* = 0.015). Vitamin B_1_–associated CpG sites were located near genes involved in DNA replication and repair, including *PRKDC* and *MCM4*. Vitamin B_2_ was associated with a CpG site within *ABLIM1*, which is involved in cytoskeletal organization. For vitamin B_3_, CpG sites were identified near genes related to immune and neuronal functions, including *SLC1A1* and *HERC6*. Notably, cg09522018 within *TXLNB* and *CITED2* was associated with both vitamin B_3_ and vitamin B_6_ intake. Vitamin B_6_-associated CpG sites were also located near genes involved in protein regulation and methylation processes, including *USP15* and *LCMT1*. Vitamin E was associated with the largest number of CpG sites mapped to genes involved in transcriptional regulation, immune signaling, and metabolic pathways, including *INTS3*, *IL1RL2*, *IP6K1*, *PRKCA*, and *ADAMTSL5*. Overall, these findings suggest potential associations between dietary vitamin intake and locus-specific DNA methylation patterns, although effect sizes were modest and functional consequences remain unclear.

### 3.3. Volcano Plot of the Epigenome-Wide Association Study (EWAS) of Vitamin Intake and DNA Methylation

The volcano plots show the associations between vitamin intake and DNA methylation across all analyzed CpG sites (Figure 2). With Bonferroni correction for genome wide significance threshold, only two CpG sites (cg05064181for vitamin B_2_, and cg19831775 for vitamin B_6_) reached genome-wide significance. Notably for vitamin E, a greater number of significant CpG sites displayed negative β values than positive β values, suggesting that higher intake of vitamins E was more frequently associated with decreased DNA methylation (hypomethylation) than increased DNA methylation (hypermethylation) at these loci. These findings identify candidate CpG sites that may play important roles in vitamin E-related epigenetic regulation and warrant further investigation.

### 3.4. Vitamin Intake and DNA Methylation at CpG Sites Stratified by Race

In race-stratified analyses, several CpG sites demonstrated consistent directions of association across African American and European American participants; however, statistical significance was attenuated in subgroup analyses, as shown in Table 3. No CpG sites remained statistically significant after FDR correction in Black participants. In White participants, several CpG sites remained significant at the predefined FDR threshold, including cg24563317, associated with vitamin B_1_ intake, and cg19831775, associated with vitamin B_6_ intake. Differences between racial groups may reflect reduced statistical power, variability in dietary exposure, or population-specific heterogeneity. In addition, the observed racial differences in vitamin intake may reflect variations in dietary patterns, food preferences, socioeconomic factors, and supplement use. Future studies examining dietary patterns and nutrient sources may help clarify these differences and their potential influence on DNA methylation. Therefore, subgroup findings should be interpreted cautiously. Overall, the results demonstrate significant associations between vitamin intake and methylation at multiple CpG sites. Effect size was generally consistent in direction across ancestries, but effect magnitudes varied between Whites and Blacks (Supplementary Table S1).

### 3.5. Sex-Stratified Associations Between Vitamin Intake and DNA Methylation at Specific CpG Sites

We examined the association stratified by sex between dietary intake of vitamins B_1_, B_2_, B_3_, B_6_, and E with blood DNA methylation levels at individual CpG sites. Among the analyzed CpG sites, only cg19831775 showed a highly significant association with vitamin B_6_, particularly in female participants. It exhibited a *p* value of 3.50 × 10^−9^ and an FDR of 0.003, indicating a link between vitamin B_6_ intake and methylation changes at this site. While several CpG sites showed *p* values at the border of significance, such as those associated with vitamins B_1_, B_3_, and E, none reached the same level of statistical significance with FDR_*p* ≤ 0.1. Overall, these results suggest no strong evidence of sex-specific differences (Supplementary Table S2).

### 3.6. Recommended Dietary Allowances (RDA) for Selected Micronutrients According to the NIH Office of Dietary Supplements

Recommended Dietary Allowances (RDA) were obtained from the NIH Office of Dietary Supplements. As shown in Table 4, sex-specific RDA values were applied where appropriate for vitamin A and B-complex vitamins (B_1_, B_2_, B_3_, and B_6_), while unified RDA values were used for vitamins E and B_12_ due to identical recommendations across sexes. Vitamin A intake was expressed as retinol activity equivalents (µg RAE/day), vitamin E as α-tocopherol (mg/day), niacin as niacin equivalents (mg NE/day), and vitamins B_1_, B_2_, and B_6_ in mg/day. Vitamin B_12_ intake was expressed in micrograms (µg/day). These reference values were used for the biological interpretation of exposure distributions rather than as analytical cutoffs in epigenome-wide association models.

## 4. Discussion

In this exploration of the epigenome-wide association study within the Southern Community Cohort Study (SCCS), we identified modest associations between dietary intake of vitamins B_1_, B_2_, B_3_, B_6_, and E and DNA methylation at 34 CpG sites in a predominantly low-income, racially diverse population. These findings extend nutritional epigenetics research to underserved populations that are often underrepresented in molecular epidemiology but experience a high burden of chronic disease [38]. The observed associations were locus-specific and generally small in magnitude, and no functional validation was performed; therefore, biological interpretation remains tentative.

Associations were strongest for vitamins B_6_ and E, followed by B_1_, B_2_, and B_3_, whereas vitamins A and B_12_ show no statistically significant CpG-specific associations. These patterns are consistent with the known biological roles of these micronutrients in one-carbon metabolism, redox balance, and enzymatic regulation, suggesting that these vitamins may be associated with DNA methylation patterns through mechanisms related to methyl group availability, oxidative stress, and cofactor-dependent enzymatic activity. Importantly, the identified associations were locus-specific rather than global, suggesting that these vitamins may be associated with susceptible genomic regions involved in immune regulation, metabolic homeostasis, and cellular stress responses [9].

Our gene-level findings in Table 2 further supported potential biological relevance. Vitamin B_1_-associated CpG sites were linked to genes involved in DNA repair [23] and glycoprotein processing; vitamin B_2_ was associated with *ABLIM1*, a gene related to cytoskeletal organization; vitamin B_3_ was associated with immune- and redox-related pathways [39]; and vitamin B_6_ was associated with loci involved in TGF-β signaling, ubiquitination, and post-transcriptional regulation [8,40,41]. Vitamin E showed the largest number of CpG associations, mapping to genes involved in immune modulation, lipid metabolism, and antioxidant defense [42]. In addition, overlapping CpG associations between vitamins B_3_ and B_6_ may reflect interdependence between one-carbon metabolism and redox-related pathways. However, because no functional validation was performed, the biological significance of these methylation differences remains uncertain and requires further experimental confirmation.

The observed racial differences in vitamin intake may reflect variations in dietary patterns, food preferences, socioeconomic factors, and supplement use. Future studies examining dietary patterns may help clarify these differences.

Sex-stratified analyses demonstrated generally consistent directions of association across African American and European American participants and between men and women, although statistical significance was attenuated in subgroup analyses. The stronger association observed between vitamin B_6_ intake and cg19831775 in women may indicate potential sex-specific metabolic or hormonal modulation; however, no robust race-specific interactions were identified in our dataset. Differences in statistical significance between subgroup analyses may also reflect reduced statistical power and heterogeneity in dietary exposure distributions.

From a clinical perspective, the identified vitamin-associated methylation changes may contribute to the understanding of biological pathways involved in immune regulation, metabolic health, and chronic disease susceptibility. Although the observed effect were modest and causal relationships cannot be inferred, these findings support the possibility that long-term dietary habits may influence epigenetic regulation and potentially contribute to disease risk. Future prospective studies are needed to determine whether these methylation differences have clinical significance.

Other factors beyond dietary vitamin intake may influence DNA methylation patterns, including medication use, antibiotic exposure, and other therapeutic interventions, and therefore represent potential confounding factors. Although information on some of these variables was available, they were not included as covariates because they were considered randomly distributed within this large population-based cohort. This may have resulted in residual confounding and should be considered when interpreting our findings. Future studies should incorporate detailed medication and treatment histories to further clarify their effects on vitamin-related DNA methylation.

The SCCS provides several important strengths, including a relatively large sample size, substantial socioeconomic diversity, and detailed dietary assessment within an understudied population [29,30,31]. In addition, the use of the Illumina Infinium MethylationEPIC v2.0 BeadChip enabled high-resolution assessment of genome-wide DNA methylation patterns across approximately 930,000 CpG sites.

Several limitations should be acknowledged. First, the cross-sectional design precludes conclusions regarding temporality or causality. Second, dietary intake was estimated using Food Frequency Questionnaires (FFQs), which are subject to recall bias and measurement errors. Although the SCCS FFQ was previously validated for this population, exposure misclassification remains possible and may have attenuated the observed associations toward the null [43,44]. Third, we applied an FDR_*p* threshold of <0.1 to balance discovery in an epigenome-wide context; however, this relatively permissive threshold increases the possibility of false-positive findings and warrants cautious interpretation. Fourth, no independent replication cohort was available to validate the observed associations, limiting external generalizability. Finally, methylation was measured in peripheral blood, which may not fully reflect tissue-specific epigenetic variation relevant to disease-related biological processes.

Overall, our findings provide hypothesis-generating evidence that dietary intake of vitamins B_1_, B_2_, B_3_, B_6_, and E may be associated with modest, locus-specific differences in DNA methylation. Although the evidence of association was generally weak, and the biological significance remains uncertain, these results contribute to the growing literature on nutritional epigenetics in underrepresented populations. Future longitudinal, mechanistic, and replication studies are needed to clarify temporality, reproducibility, and the functional relevance of micronutrient-associated epigenetic variation in chronic disease risk and health disparities.

## 5. Conclusions

In this exploration epigenome-wide association study, we identified modest associations between dietary intake of vitamins B_1_, B_2_, B_3_, B_6_, and E and DNA methylation at 34 CpG sites in a diverse cohort. These associations were locus-specific and generally small in magnitude, and no functional validation was performed; therefore, biological interpretation remains tentative. The absence of replication and reliance on self-reported dietary intake further limit inference. Nonetheless, these findings provide hypothesis-generating evidence that dietary micronutrients may be associated with epigenetic variation at specific genomic regions, warranting confirmation in longitudinal and mechanistic studies.

## Figures and Tables

**Table 1:** Selected characteristics of participants from the Southern Community Cohort Study (SCCS), stratified by race.

Characteristics	All (n = 2030)	Blacks (n = 1108)	Whites (n = 922)	*p* ‡
Age at baseline, years, median (IQR)	54 (48–61)	52 (47–60)	57 (49–63)	<0.001
Males, n (%)	934 (46.0)	575 (51.9)	359 (38.9)	<0.001
Education, n (%) †				0.004
<High school	776 (38.3)	459 (41.4)	317 (34.4)	
High school	679 (33.5)	344 (31.1)	335 (36.4)	
Some college	449 (22.1)	246 (22.2)	203 (22.0)	
≥College	125 (6.2)	59 (5.3)	66 (7.2)	
Annual household income, n (%) †				<0.001
<$15,000	1290 (64.1)	741 (67.5)	549 (60.0)	
$15,000–<$25,000	440 (21.9)	241 (22.0)	199 (21.8)	
$25,000–<$50,000	213 (10.6)	92 (8.4)	121 (13.2)	
≥$50,000	70 (3.5)	24 (2.2)	46 (5.0)	
BMI, kg/m^2^, n (%) †				<0.001
<25	651 (32.3)	401 (36.5)	250 (27.3)	
25–<30	661 (32.8)	347 (31.6)	314 (34.3)	
30–<35	382 (19.0)	187 (17.0)	195 (21.3)	
≥35	320 (15.9)	164 (14.9)	156 (17.1)	
Smoking status, n (%) †				<0.001
Never	137 (6.8)	108 (9.8)	29 (3.2)	
Former	480 (23.7)	230 (20.8)	250 (27.1)	
Current	1413 (69.6)	770 (69.5)	643 (69.7)	
Diabetes history, n (%) †	384 (18.9)	204 (18.4)	180 (19.5)	0.524
Pack-years (ever smokers), median (IQR)	24.0 (11.9–41.0)	17.0 (9.2–29.0)	34.5 (19.0–50.0)	<0.001
Alcohol (drinks/day), median (IQR)	0 (0–0.7)	0.09 (0–1.4)	0 (0–0.2)	<0.001
Total energy intake (kcal/day), median (IQR)	2155.1 (1519.5–3165.2)	2395.3 (1642.1–3522.0)	1925.9 (1408.7–2731.9)	<0.001
Physical activity (MET-h/day), median (IQR)	16.9 (8.6–30.4)	17.2 (8.8–30.3)	16.6 (8.5–30.9)	0.249
Healthy Eating Index, median (IQR)	55.2 (47.1–64.0)	55.6 (48.1–63.6)	54.6 (46.6–64.4)	0.306
Vitamin A (µg/day), median (IQR)	775.1 (525.9–1129.2)	831.5 (555.6–1264.1)	726.4 (498.2–997.3)	<0.001
Vitamin B1 (mg/day), median (IQR)	1.7 (1.2–2.4)	1.8 (1.2–2.6)	1.6 (1.1–2.2)	<0.001
Vitamin B2 (mg/day), median (IQR)	2.3 (1.6–3.2)	2.2 (1.5–3.2)	2.4 (1.7–3.2)	<0.001
Vitamin B3 (mg/day), median (IQR)	24.8 (17.2–36.7)	26.7 (17.8–39.8)	22.8 (16.6–33.5)	<0.001
Vitamin B6 (mg/day), median (IQR)	2.0 (1.3–3.0)	2.1 (1.4–3.2)	1.8 (1.2–2.7)	<0.001
Vitamin B12 (µg/day), median (IQR)	5.7 (3.7–9.1)	6.1 (3.8–10.1)	5.3 (3.5–8.1)	<0.001
Vitamin E (mg/day), median (IQR)	7.3 (5.0–10.7)	7.7 (5.3–11.3)	6.9 (4.8–9.6)	<0.001

† Categories may not sum to total due to missing data. ‡ *p*-values based on Wilcoxon rank-sum tests (continuous variables) and chi-square tests (categorical variables).

**Table 2:** Associations between vitamin intake (B_1_, B_2_, B_3_, B_6_, E) and DNA methylation at CpG sites.

Vitamin	CpG Site	CHR *	Gene	Region	CpG Position	Beta	Se	*p* Value	FDR_*p*
B1	cg15431659	3	*XXYLT1*	Intron	195,180,628	−0.113	0.022	2.98 × 10^−7^	0.082
B1	cg24563317	8	*MCM4*	Exon	4,7960,262	0.107	0.021	1.94 × 10^−7^	0.082
B1	cg18360612	12	—	—	130,880,199	−0.122	0.024	2.87 × 10^−7^	0.082
B2	cg05064181	10	*ABLIM1*	Intron	139,323,734	−0.166	0.03	6.04 × 10^−8^	0.05
B3	cg15881994	4	*HERC6*	Exon	88,379,004	0.132	0.026	3.25 × 10^−7^	0.091
B3	cg09522018	6	—	—	139,323,734	0.132	0.025	1.65 × 10^−7^	0.091
B3	cg17599943	9	*SLC1A1*	Exon	4,585,465	−0.215	0.042	3.31 × 10^−7^	0.091
B6	cg20525831	1	*ANKRD35*	TSS	145,885,871	0.102	0.021	6.41 × 10^−7^	0.085
B6	cg19775958	3	*CACNA1D*	Intron	53,748,967	−0.097	0.02	7.25 × 10^−7^	0.085
B6	cg09522018	6	—	—	139,323,734	0.098	0.019	4.24 × 10^−7^	0.085
B6	cg14361907	9	*RAPGEF1*	Intron	131,610,278	−0.139	0.027	3.97 × 10^−7^	0.085
B6	cg19831775	12	—	—	107,660,902	−0.111	0.0197	1.86 × 10^−8^	0.015
B6	cg20201833	12	*USP15*	Intron	62,260,937	0.09	0.018	7.20 × 10^−7^	0.085
B6	cg01452189	16	*LCMT1*	Exon	25,178,105	−0.153	0.03	4.62 × 10^−7^	0.085
E	cg26904702	1	*INTS3*	Exon	153,774,423	−0.112	0.024	1.92 × 10^−6^	0.088
E	cg17571972	1	*NSL1*	Exon	212,728,746	−0.089	0.019	1.50 × 10^−6^	0.088
E	cg04435410	1	*LINC01648*	Flanking	30,087,890	−0.157	0.03	1.68 × 10^−7^	0.046
E	cg00066041	1	—	—	2,575,640	−0.143	0.03	1.69 × 10^−6^	0.088
E	cg02560110	2	*ABLIM1*, *DNMT3A*	Intron	25,253,251	−0.084	0.016	1.39 × 10^−7^	0.046
E	cg16720946	2	*LINCO1126*	Exon	43,227,071	−0.172	0.036	1.51 × 10^−6^	0.088
E	cg24394667	2	*IL1RL2*	Promoter	102,186,608	−0.163	0.034	1.62 × 10^−6^	0.088
E	cg04641912	3	*IP6K1*	Promoter	49,724,290	0.11	0.023	1.05 × 10^−6^	0.088
E	cg00187987	5	*MBLAC2*	Exon	90,474,700	0.083	0.017	7.65 × 10^−7^	0.088
E	cg07669182	5	*FYB1*	Intron	39,203,653	−0.077	0.016	1.94 × 10^−6^	0.088
E	cg13763524	5	—	—	75,735,039	−0.165	0.035	2.42 × 10^−6^	0.099
E	cg03613618	8	*CPQ*	Intron	96,646,097	−0.095	0.02	2.34 × 10^−6^	0.099
E	cg02448244	9	—	—	36,739,125	−0.16	0.032	7.85 × 10^−7^	0.088
E	cg20580508	10	—	—	8,656,933	−0.177	0.037	1.74 × 10^−6^	0.088
E	cg20761187	11	*LINC02730*	Intron	134,031,029	−0.151	0.031	1.00 × 10^−6^	0.088
E	cg00130246	11	*ACCS*	Exon	43,227,071	−0.172	0.036	1.51 × 10^−6^	0.088
E	cg01936636	11	*CARS1*	Intron	3,037,216	−0.118	0.025	2.55 × 10^−6^	0.1
E	cg22786316	15	—	—	70,560,829	−0.084	0.016	1.67 × 10^−7^	0.046
E	cg24818690	16	—	—	48,613,144	−0.084	0.017	1.35 × 10^−6^	0.088
E	cg03276430	17	*PRKCA*	Intron	66,567,336	−0.129	0.027	1.78 × 10^−6^	0.088
E	cg16425342	19	*ADAMTSL5*	Intron	1,509,776	−0.101	0.021	1.02 × 10^−6^	0.088

* CHR: Chromosome, Gene: gene near the CpG site, Beta: effect estimate (positive indicating increase and negative indicating decrease), Se: standard error, FDR-*p*: false discovery rate adjusted *p*-value.

**Table 3:** Associations between dietary vitamin intake and blood methylation at CpG sites by race.

		Whites (EA)			Blacks (AA)		
Vitamin	CpG Site	Beta	Se	FDR_*p*	Beta	Se	FDR_*p*
B1	cg15431659	−0.159	0.033	0.043	−0.084	0.029	0.956
B1	cg18360612	−0.131	0.035	0.062	−0.121	0.032	0.956
B1	cg24563317	0.161	0.029	0.02	0.069	0.029	0.956
B3	cg09522018	0.146	0.037	0.051	0.118	0.035	0.843
B3	cg15881994	0.126	0.038	0.075	0.136	0.037	0.843
B3	cg17599943	−0.188	0.062	0.095	−0.245	0.057	0.843
B6	cg19831775	−0.103	0.025	0.089	−0.118	0.030	0.674
B6	cg20525831	0.119	0.029	0.089	0.088	0.029	0.674
E	cg02560110	−0.111	0.023	0.086	−0.064	0.021	0.284
E	cg02448244	−0.210	0.044	0.086	−0.106	0.047	0.381

**Table 4:** NIH Recommended Dietary Allowances (RDA) for vitamins evaluated in the EWAS.

Vitamin	RDA Male	RDA Female	Unit
Vitamin A	900	700	µg RAE/day
Vitamin E	15	15	mg α-tocopherol/day
Vitamin B1 (Thiamin)	1.2	1.1	mg/day
Vitamin B2 (Riboflavin)	1.3	1.1	mg/day
Vitamin B3 (Niacin)	16	14	mg NE/day
Vitamin B6 (Pyridoxine)	1.3–1.7	1.3–1.5	mg/day
Vitamin B12 (Cobalamin)	2.4	2.4	µg/day

## Data Availability

The data presented in this study are available on request from the corresponding author due to privacy or ethical restrictions.

## Supplementary Materials

The following supporting information can be downloaded at: https://www.mdpi.com/article/10.3390/nu18152474/s1, Supplementary Table S1: Associations Between Vitamin Intake (B_1_, B_2_, B_3_, B_6_, E) and DNA Methylation at CpG Sites stratified by Race; Supplementary Table S2: Associations Between Vitamin Intake (B_1_, B_2_, B_3_, B_6_, E) and DNA Methylation at CpG Sites Stratified by Sex.

## Abbreviations

BMI	Body Mass Index
SE	Standard Error of the Beta estimate
FDR	False Discovery Rate
IQR	Interquartile Range
SCCS	Southern Community Cohort Study
FFQ	Food Frequency Questionnaire(s)
CHC	Community Health Centers
USDA	United States Department of Agriculture
