# Supplementary material for: Nutritional Epigenetics of Vitamin Intake (A, B1, B2, B3, B6, B12, and E): Associations with Genome-Wide DNA Methylation
Source: Nutrients. 2026 Jul 30;18(15):2474. doi: 10.3390/nu18152474 (PMC13467880; doi:10.3390/nu18152474)
Supplement: Supplementary file 1 [file nutrients-18-02474-s001.zip › nutrients-4381609-supplementary.pdf]

SupplementaryTable S1: Associations Between Vitamin Intake (B1, B2, B3, B6, E) and DNA Methylation at CpG Sites Stratefied by Race

		All participants				Whites (European Americans)			Blacks (African Americans)			
Vitamin	CpG-site	Beta	Se	*p* value	FDR	Beta	Se	*p* value	Beta	Se	*p* value	*p* _interaction
B1	cg15431659	-0.113	0.022	2.98E-07	0.082	-0.159	0.033	2.21E-06	-0.084	0.029	3.26E-03	0.126
B1	cg18360612	-0.122	0.024	2.87E-07	0.082	-0.131	0.035	1.68E-04	-0.121	0.032	2.07E-04	0.999
B1	cg24563317	0.107	0.021	1.94E-07	0.082	0.161	0.029	4.96E-08	0.069	0.029	1.89E-02	0.639
B2	cg05064181	-0.166	0.030	6.04E-08	0.050	-0.187	0.047	8.33E-05	-0.143	0.042	6.06E-04	0.242
B3	cg09522018	0.132	0.025	1.65E-07	0.091	0.146	0.037	9.84E-05	0.118	0.035	8.59E-04	0.289
B3	cg15881994	0.132	0.026	3.25E-07	0.091	0.126	0.038	9.06E-04	0.136	0.037	2.78E-04	0.918
B3	cg17599943	-0.215	0.042	3.31E-07	0.091	-0.188	0.062	2.41E-03	-0.245	0.057	2.00E-05	0.696
B6	cg01452189	-0.153	0.030	4.62E-07	0.085	-0.136	0.042	1.39E-03	-0.175	0.044	6.36E-05	0.684
B6	cg09522018	0.098	0.019	4.24E-07	0.085	0.099	0.028	3.73E-04	0.092	0.027	8.37E-04	0.360
B6	cg14361907	-0.139	0.027	3.97E-07	0.085	-0.099	0.037	7.32E-03	-0.165	0.040	3.13E-05	0.501
B6	cg19831775	-0.111	0.020	1.86E-08	0.015	-0.103	0.025	4.14E-05	-0.118	0.030	1.02E-04	0.654
B6	cg19775958	-0.097	0.020	7.25E-07	0.085	-0.068	0.029	2.15E-02	-0.131	0.026	3.90E-07	0.053
B6	cg20201833	0.090	0.018	7.20E-07	0.085	0.079	0.025	1.26E-03	0.104	0.026	7.80E-05	0.252
B6	cg20525831	0.102	0.021	6.41E-07	0.085	0.119	0.029	3.39E-05	0.088	0.029	2.74E-03	0.623
E	cg02560110	-0.084	0.016	1.39E-07	0.046	-0.111	0.023	9.77E-07	-0.064	0.021	2.75E-03	0.370
E	cg22786316	-0.084	0.016	1.67E-07	0.046	-0.083	0.020	2.93E-05	-0.091	0.025	3.35E-04	0.352
E	cg04435410	-0.157	0.030	1.68E-07	0.046	-0.172	0.043	7.13E-05	-0.149	0.043	5.12E-04	0.339
E	cg16720946	0.106	0.021	6.78E-07	0.088	0.100	0.030	7.80E-04	0.119	0.031	1.11E-04	0.216
E	cg00187987	0.083	0.017	7.65E-07	0.088	0.067	0.024	4.63E-03	0.101	0.023	1.37E-05	0.338
E	cg02448244	-0.160	0.032	7.85E-07	0.088	-0.210	0.044	1.84E-06	-0.106	0.047	2.51E-02	0.377
E	cg20761187	-0.151	0.031	1.00E-06	0.088	-0.163	0.043	1.41E-04	-0.142	0.044	1.30E-03	0.300
E	cg16425342	-0.101	0.021	1.02E-06	0.088	-0.090	0.027	8.98E-04	-0.115	0.032	3.29E-04	0.388
E	cg04641912	0.110	0.023	1.05E-06	0.088	0.096	0.032	2.86E-03	0.125	0.032	7.84E-05	0.512
E	cg24818690	-0.084	0.017	1.35E-06	0.088	-0.080	0.022	2.34E-04	-0.094	0.027	4.70E-04	0.127
E	cg17571972	-0.089	0.019	1.50E-06	0.088	-0.079	0.022	2.74E-04	-0.115	0.030	1.53E-04	9.45E-04
E	cg00130246	-0.172	0.036	1.51E-06	0.088	-0.175	0.052	7.67E-04	-0.153	0.048	1.46E-03	0.787
E	cg24394667	-0.163	0.034	1.62E-06	0.088	-0.162	0.046	5.00E-04	-0.157	0.052	2.54E-03	0.702
E	cg00066041	-0.143	0.030	1.69E-06	0.088	-0.156	0.044	3.77E-04	-0.132	0.040	1.18E-03	0.855
E	cg20580508	-0.177	0.037	1.74E-06	0.088	-0.162	0.054	2.67E-03	-0.186	0.051	2.56E-04	0.612
E	cg03276430	-0.129	0.027	1.78E-06	0.088	-0.103	0.037	5.39E-03	-0.171	0.040	2.49E-05	0.144
E	cg26904702	-0.112	0.024	1.92E-06	0.088	-0.109	0.034	1.36E-03	-0.107	0.034	1.51E-03	0.607
E	cg07669182	-0.077	0.016	1.94E-06	0.088	-0.055	0.025	2.63E-02	-0.102	0.022	3.66E-06	0.114
E	cg03613618	-0.095	0.020	2.34E-06	0.099	-0.098	0.028	4.92E-04	-0.103	0.029	3.63E-04	0.215
E	cg13763524	-0.165	0.035	2.42E-06	0.099	-0.199	0.053	1.67E-04	-0.126	0.048	8.59E-03	0.203
E	cg01936636	-0.118	0.025	2.55E-06	0.100	-0.167	0.037	6.90E-06	-0.079	0.035	2.54E-02	0.556

SupplementaryTable S2: Associations Between Vitamin Intake (B1, B2, B3, B6, E) and DNA Methylation at CpG Sites Stratefied by Sex

		All participants				Male			Female			
Vitamin	CpG-site	Beta	Se	*p* value	FDR	Beta	Se	*p* value	Beta	Se	*p* value	*p* _interaction
B1	cg15431659	-0.113	0.022	2.98E-07	0.082	-0.117	0.028	4.11E-05	-0.111	0.031	3.94E-04	0.411
B1	cg18360612	-0.122	0.024	2.87E-07	0.082	-0.120	0.040	2.83E-03	-0.114	0.031	1.95E-04	0.549
B1	cg24563317	0.107	0.021	1.94E-07	0.082	0.129	0.035	2.41E-04	0.092	0.026	4.10E-04	0.750
B2	cg05064181	-0.166	0.030	6.04E-08	0.050	-0.182	0.056	1.26E-03	-0.148	0.037	8.08E-05	0.945
B3	cg09522018	0.132	0.025	1.65E-07	0.091	0.116	0.043	7.05E-03	0.133	0.034	8.79E-05	0.395
B3	cg15881994	0.132	0.026	3.25E-07	0.091	0.106	0.043	1.38E-02	0.140	0.034	4.67E-05	0.225
B3	cg17599943	-0.215	0.042	3.31E-07	0.091	-0.229	0.070	1.04E-03	-0.210	0.054	1.07E-04	0.674
B6	cg01452189	-0.153	0.030	4.62E-07	0.085	-0.142	0.046	2.30E-03	-0.161	0.041	8.16E-05	0.938
B6	cg09522018	0.098	0.019	4.24E-07	0.085	0.072	0.032	2.80E-02	0.106	0.025	2.28E-05	0.155
B6	cg14361907	-0.139	0.027	3.97E-07	0.085	-0.148	0.041	2.91E-04	-0.132	0.038	4.90E-04	0.591
B6	cg19831775	-0.111	0.020	1.86E-08	0.015	-0.060	0.034	7.46E-02	-0.142	0.024	3.50E-09	0.193
B6	cg19775958	-0.097	0.020	7.25E-07	0.085	-0.083	0.030	6.57E-03	-0.106	0.026	5.87E-05	0.723
B6	cg20201833	0.090	0.018	7.20E-07	0.085	0.081	0.032	1.07E-02	0.093	0.022	2.98E-05	0.254
B6	cg20525831	0.102	0.021	6.41E-07	0.085	0.086	0.032	8.03E-03	0.102	0.027	1.53E-04	0.059
E	cg02560110	-0.084	0.016	1.39E-07	0.046	-0.069	0.025	6.68E-03	-0.086	0.021	5.38E-05	0.078
E	cg22786316	-0.084	0.016	1.67E-07	0.046	-0.128	0.027	3.29E-06	-0.069	0.020	5.92E-04	0.038
E	cg04435410	-0.157	0.030	1.68E-07	0.046	-0.195	0.046	2.85E-05	-0.123	0.039	1.76E-03	0.391
E	cg16720946	0.106	0.021	6.78E-07	0.088	0.090	0.035	1.05E-02	0.115	0.027	2.36E-05	0.179
E	cg00187987	0.083	0.017	7.65E-07	0.088	0.087	0.026	7.82E-04	0.074	0.022	7.20E-04	0.091
E	cg02448244	-0.160	0.032	7.85E-07	0.088	-0.215	0.054	8.40E-05	-0.117	0.042	5.53E-03	0.267
E	cg20761187	-0.151	0.031	1.00E-06	0.088	-0.246	0.050	1.07E-06	-0.113	0.041	5.63E-03	0.071
E	cg16425342	-0.101	0.021	1.02E-06	0.088	-0.142	0.034	4.01E-05	-0.070	0.026	8.24E-03	0.853
E	cg04641912	0.110	0.023	1.05E-06	0.088	0.141	0.035	6.44E-05	0.090	0.030	2.87E-03	0.928
E	cg24818690	-0.084	0.017	1.35E-06	0.088	-0.129	0.030	1.52E-05	-0.062	0.022	5.30E-03	0.161
E	cg17571972	-0.089	0.019	1.50E-06	0.088	-0.111	0.033	8.27E-04	-0.068	0.022	2.52E-03	0.314
E	cg00130246	-0.172	0.036	1.51E-06	0.088	-0.209	0.056	2.23E-04	-0.156	0.046	6.87E-04	0.546
E	cg24394667	-0.163	0.034	1.62E-06	0.088	-0.089	0.047	5.98E-02	-0.189	0.046	4.05E-05	0.010
E	cg00066041	-0.143	0.030	1.69E-06	0.088	-0.102	0.045	2.34E-02	-0.165	0.039	3.04E-05	0.061
E	cg20580508	-0.177	0.037	1.74E-06	0.088	-0.213	0.060	4.44E-04	-0.159	0.047	7.48E-04	0.233
E	cg03276430	-0.129	0.027	1.78E-06	0.088	-0.084	0.047	7.37E-02	-0.154	0.034	7.14E-06	0.255
E	cg26904702	-0.112	0.024	1.92E-06	0.088	-0.121	0.039	1.86E-03	-0.102	0.030	6.79E-04	0.098
E	cg07669182	-0.077	0.016	1.94E-06	0.088	-0.087	0.028	2.02E-03	-0.082	0.020	3.80E-05	0.399
E	cg03613618	-0.095	0.020	2.34E-06	0.099	-0.073	0.033	2.91E-02	-0.110	0.026	2.05E-05	0.730
E	cg13763524	-0.165	0.035	2.42E-06	0.099	-0.195	0.056	5.27E-04	-0.159	0.046	5.32E-04	0.462
E	cg01936636	-0.118	0.025	2.55E-06	0.100	-0.117	0.041	4.36E-03	-0.124	0.032	1.08E-04	0.830
